# Seed Weight and Trade-Offs: An Experiment in False Rhodes Grasses under Different Aridity Conditions

**DOI:** 10.3390/plants11212887

**Published:** 2022-10-28

**Authors:** Lorena Marinoni, Juan M. Zabala, R. Emiliano Quiroga, Geraldina A. Richard, José F. Pensiero

**Affiliations:** 1 Instituto de Ciencias Agropecuarias del Litoral (ICiAgro Litoral UNL-CONICET), Kreder 2805, Esperanza 3080, Argentina; 2 Facultad de Ciencias Agrarias, Universidad Nacional del Litoral (FCA-UNL), Kreder 2805, Esperanza 3080, Argentina; 3 Instituto Nacional de Tecnología Agropecuaria, EEA Catamarca, Sumalao 4705, Argentina; 4 Cátedra de Manejo de Pastizales Naturales, Facultad de Ciencias Agrarias, Universidad Nacional de Catamarca, Catamarca 4700, Argentina

**Keywords:** germination, plant functional traits, *Leptochloa crinita*, *L. pluriflora*, local adaptation

## Abstract

The false Rhodes grasses [*Leptochloa crinita* (Lag.) P.M. Peterson and N.W. Snow and *Leptochloa pluriflora* (E. Fourn.) P.M. Peterson and N.W. Snow] are considered valuable native forage resources for arid and semiarid rangelands in Argentina and the United States. Effectively using plant materials as forage under aridity conditions requires understanding their resource allocation under those conditions. In the present study, plant functional traits were evaluated in six populations of each false Rhodes grass species from different geographic origin in a humid and an arid region. The evaluation was focused on seed weight, due to the key role of this trait in plant survival. The implication of seed weight in germination under osmotic stress and trade-off relationships between functional traits were also analysed. A fixed ontogenetic variation was found in both species, since populations maintained a stable seed weight across environments. The tolerance to osmotic stress at germination stage was more related to seed weight than to population origin or maternal environment of seeds; heavier-seeded populations produced heavier seedlings instead of a higher number of germinated seeds or higher germination rates. Some traits varied between environments but other traits exhibited a fixed response. Variation patterns among populations were similar within environments and in some cases even for populations from the same geographic origin, revealing a fixed ontogenetic variation; this phenomenon was clearer in *L. crinita* than in *L. pluriflora*. Moreover, several different trade-off strategies were detected in both species. These results reinforce the knowledge about the key role of seed weight in survival and performance of seedlings at initial growth stages under arid conditions; however, at advanced stages, other traits would have an important function in growth and development of false Rhodes grasses.

## 1. Introduction

Arid and semiarid lands cover more than half of the Earth’s land surface, including Argentina, where these lands support sheep, goats and parts of cattle production [1,2]. These lands harbour a broad natural ecological variation, with vegetation being usually a combination of herbaceous and woody species [1]. Those native species have undergone a long process of natural selection and adaptation to the local environment; in addition, they are potentially valuable genetic resources for revegetation, rehabilitation or enrichment of arid and semiarid rangelands that have been degraded, for example, by overgrazing [3,4,5,6].

Native plant species respond to environmental variation across their distribution range through genetic adaptations that are expressed in traits [7,8]. Selection mediated by natural agents and artificial selection might often act in opposite directions, and this is particularly important in native species [9]. Traits such as plant height, leaf area (or specific leaf area), stem size and seed weight have been proposed as key to understanding ecological strategies between and within species [10,11], and have been also used to explain differences in resource allocation that lead to several trade-offs. On the other hand, biomass production-related traits are the main features evaluated in forage breeding programmes [12]. These ecologically meaningful traits are considered plant functional traits, because they allow us to link morphological, biochemical, physiological, structural, phenological or behavioural characteristics to their functions. They contribute to our understanding of ecological processes and provide information for plant breeding and restoration activities [13]. Thus, identifying adaptive and allocation resource patterns in functional traits is necessary for traditional breeding of native plants [14,15,16,17], especially in stressful environments [17,18].

*Leptochloa crinita* (Lag.) P.M. Peterson and N.W. Snow and *Leptochloa pluriflora* (E. Fourn.) P.M. Peterson and N.W. Snow, known as false Rhodes grass and multiflower false Rhodes grass, respectively, are considered valuable native forage resources for arid and semiarid rangelands in Argentina and the United States [19,20,21]. Both species are warm-season perennial bunchgrasses [22]. *Leptochloa crinita* has been widely studied for restoration purposes because of its broad distribution and tolerance to extreme aridity and salinity conditions [6,15,23,24,25,26,27], whereas *L. pluriflora* has been less explored [25,26,27]. In a previous study involving populations from the entire distribution range of *L. crinita* in Argentina, cultivated in a common-garden experiment, we observed an important additive genetic component for seed weight and signs of local adaptation [26]. Heavier seeds produced heavier seedlings under both osmotic stress and non-stress conditions, conferring an advantage at initial growth stages. This positive effect of heavier seeds on germination capacity and seedling survival has been widely demonstrated [28,29,30,31]. Plant functional traits and populations with potential for plant breeding have also been identified in *L. crinita* under experimental drought conditions [15,27].

The main aim of this paper was to raise questions concerning seed weight—as a key functional trait—trade-offs with other functional traits and elucidate adaptive and/or environmental causes in such relationships. Due to the importance of the studied species as forage resources for arid environments and for restoration purposes, we asked: (a) Is seed weight more influenced by the genetic background of populations than by the environmental conditions experienced by mother plants? (b) Is the positive association between seed weight and germination stable across maternal environments and germination conditions? (c) Is the selection process in favour of high seed weight detrimental to traits related to biomass production? (d) Are differences in resource allocation among traits dependent on seed weight? Based on previous studies and background, we hypothesized that: (a) since seed weight is a moderate to high heritable trait and an adaptive trait, populations with higher seed weight would produce heavier seeds, either at a humid or an arid maternal environment; (b) germination behaviour would be driven by seed weight per se rather than maternal environment during seed development; (c) there would be trade-off relationships between seed weight and other functional traits, which would be maintained under different environmental conditions; (d) natural selection would operate in favour of heavier seeds to guarantee survival; therefore, heavier-seeded populations would allocate more resources to reproductive traits at the expense of vegetative traits, and this phenomenon would be more evident under stressful conditions (e.g., arid environments).

To answer these questions and test our predictions, we cultivated plants of six accessions of each of the two *Leptochloa* species in environments with contrasting aridity conditions during two years, using seeds collected from an unstressed common garden. We harvested seeds for seed weight and germination analyses for two consecutive years, and during the second year, we performed non-destructive and destructive measurements during vegetative and reproductive stages to evaluate functional traits. We selected accessions based on previous studies [26,27], and attempted to represent three different types of original stressful environments. For each of these environments, we included populations of intrinsically high and low seed weight. 

## 2. Results

### 2.1. Trial 1. Seed Weight and Germination Analysis

**Figure 1:**
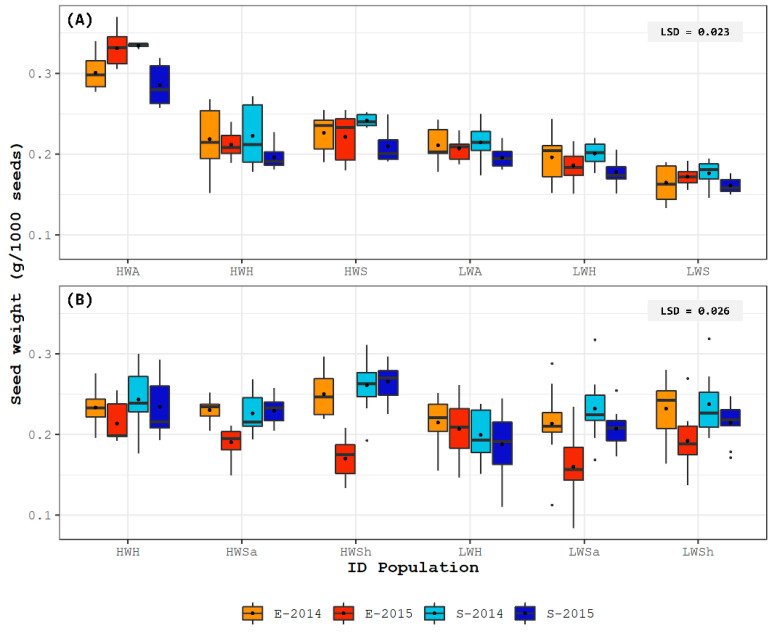
Box-plot for seed weight variation in six populations of (**A**) *Leptochloa crinita* and (**B**) *L. pluriflora,* grown in four different environments: Esperanza site, year 2014 (E-2014) and year 2015 (E-2015); Sumalao site, year 2014 (S-2014) and 2015 (S-2015). “ID populations” is composed of seed weight (high/low) and origin of population. HWA: high weight-arid origin. HWH: high weight-humid origin. HWS: high weight-saline origin. HWSa: high weight-semiarid origin. HWSh: high weight-subhumid origin. LWA: low weight-arid origin. LWH: low weight-humid origin. LWS: low weight-saline origin. LWSa: low weight-semiarid origin. LWSh: low weight-subhumid origin. LSD: Least Significant Differences according Tukey test (*p* < 0.05).

The seeds of the populations of both false Rhodes grasses maintained an almost constant weight at both experimental sites during the two consecutive growing cycles (Figure 1A,B). Seed weight of *L. crinita* in S-2015 was the lowest of all accessions; however, no interaction between populations and experimental site was found. Contrarily, for *L. pluriflora*, seed weight in E-2015 was the lowest and a significant interaction was detected (see *p*-values in Table S1). However, positive correlations were observed in both species, except for *L. pluriflora* in the E-2015 environment; such ranking mirrored the ranking in seed weight of passport data (Table 1). 

The parameters evaluated at the germination stage varied among populations within species, seed maternal environments and osmotic stress treatments, and significant interactions were found only between osmotic treatment and populations; this result may be attributed to the lack of germination of some populations under osmotic stress (see *p*-values in Table S2).

**Figure 2:**
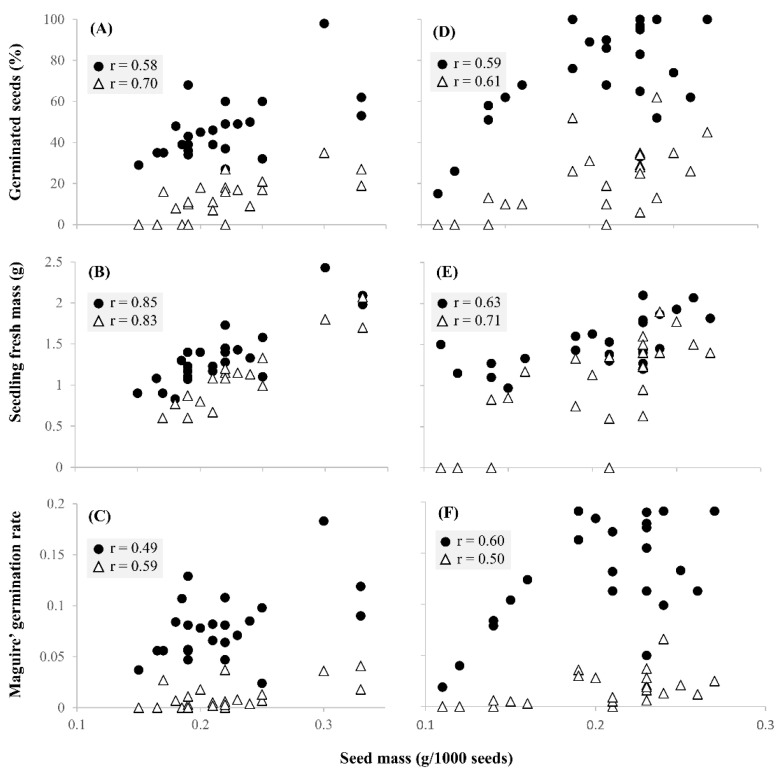
Correlation coefficients (r) of seed weight under osmotic stress (white triangles) and control conditions (black circles) with: (**A**) final emergence percentage (FEP), (**B**) seedling fresh mass (SFM) and (**C**) Maguire’ germination rate (GR) in *Leptochloa crinita*. Correlations of seed weight under osmotic stress and control conditions with: (**D**) FEP, (**E**) SFM and (**F**) GR in *Leptochloa pluriflora*.

In *L. crinita*, the low-seed weight populations from arid and saline origin did not germinate under osmotic stress (Table 2); germination of *L. pluriflora* populations harvested in Esperanza in 2015 was also affected under both non-stressful and stressful conditions (Table 3). In general, germination behaviour was more related to seed weight than to the origin of population or seed maternal environment. Moreover, the final emergence percentage (FEP), seedling fresh mass (SFM) and Maguire’s germination rate (GR) were correlated to seed weight in both species and stress treatments (*p* < 0.05), with SFM showing the strongest correlation (Figure 2B,E).

### 2.2. Trial 2. Plant Functional Traits 

**Figure 3:**
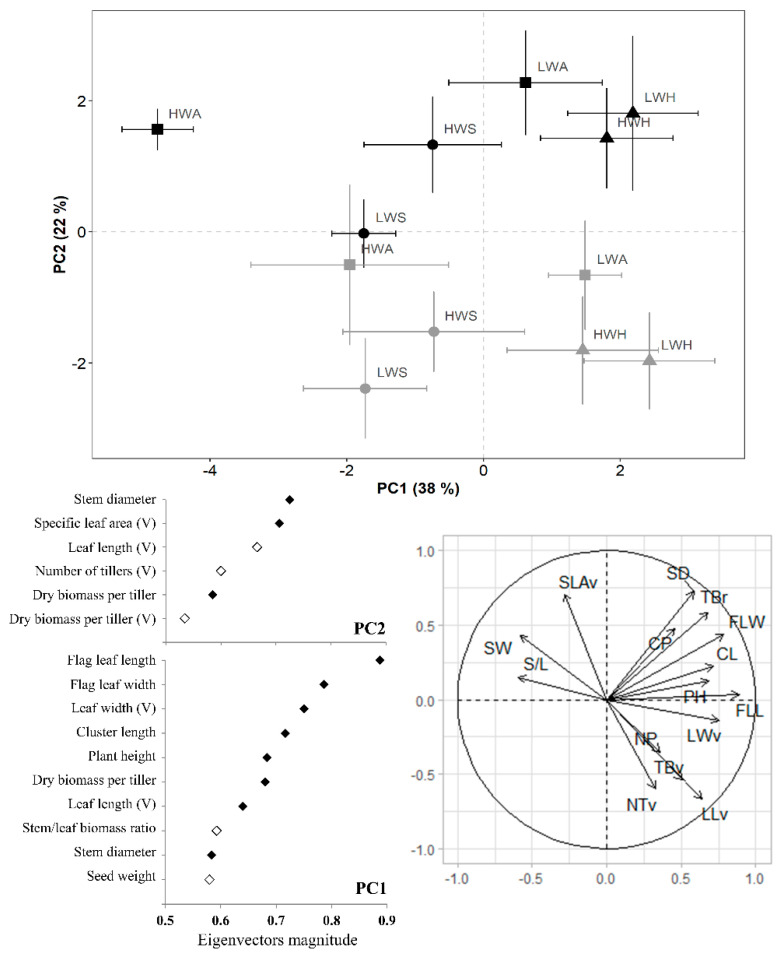
Principal Component Analysis (PCA) of plant functional traits in populations of *Leptochloa crinita* cultivated in two experimental environments: Sumalao (grey symbols) and Esperanza (black symbols) during the 2014–2015 summer season. The ID of populations is composed of seed weight (high/low) and origin of population. HWA: high weight-arid origin. HWH: high weight-humid origin. HWS: high weight-saline origin LWA: low weight-arid origin. LWH: low weight-humid origin. LWS: low weight-saline origin. PH: Plant height. NP: Number of panicles. S/L: Stem/leaf biomass ratio. TBr: tiller biomass at the reproductive stage. SW: Seed weight. FLL: Flag leaf length. FLW: Flag leaf width. CP: Number of clusters per panicle. CL: Cluster length. NTv: Number of vegetative tillers. TBv: tiller biomass at the vegetative stage. LLv: Leaf length. LWv: Leaf width. SLAv: Specific leaf area.

The PCA revealed a clear distinction between experimental environments and populations in both species. Populations were sorted according to the experimental environments along PC2 and within environments along PC1, showing the same pattern in each environment. For *L. crinita*, the first two components accounted for 60% of the total variance of the dataset. Along PC2 (22%), specific leaf area, stem diameter, number of tillers per plant, leaf length and biomass per tiller (at reproductive and vegetative stages) showed the maximum contribution to variance (Figure 3). Populations cultivated in Esperanza were located on the positive side of PC2 (opposite to those cultivated in Sumalao) due to their higher specific leaf area, stem diameter and dry biomass per tiller at the reproductive stage. Along PC1 (38%), populations were separated by features detailed in Figure 3, most of which were measured during the reproductive stage. In general, parameters measured at the vegetative stages were responsible for differences between environments, whereas traits evaluated during the reproductive stages were related to the population rank observed in both environments. Populations from humid (HWH, LWH) and saline (HWS, LWS) origins showed a similar behaviour, regardless of seed weight and experimental environment. The population HWA was very different from the other populations, with much heavier seeds, smaller leaves, fewer tillers and lower tiller biomass per plant. This result was explained by the correlations found among traits. In general, vegetative traits were grouped and correlated among them; the same result was observed for reproductive traits. Most of the reproductive traits are located in the upper right quadrant of Figure 3 and no trait is located in the lower left quadrant, revealing few negative correlations. Only seed weight, stem/leaf biomass rate and specific leaf area were negatively correlated with vegetative parameters, the latter being located in the lower right quadrant of Figure 3 (see correlation matrix in Table S3). 

**Figure 4:**
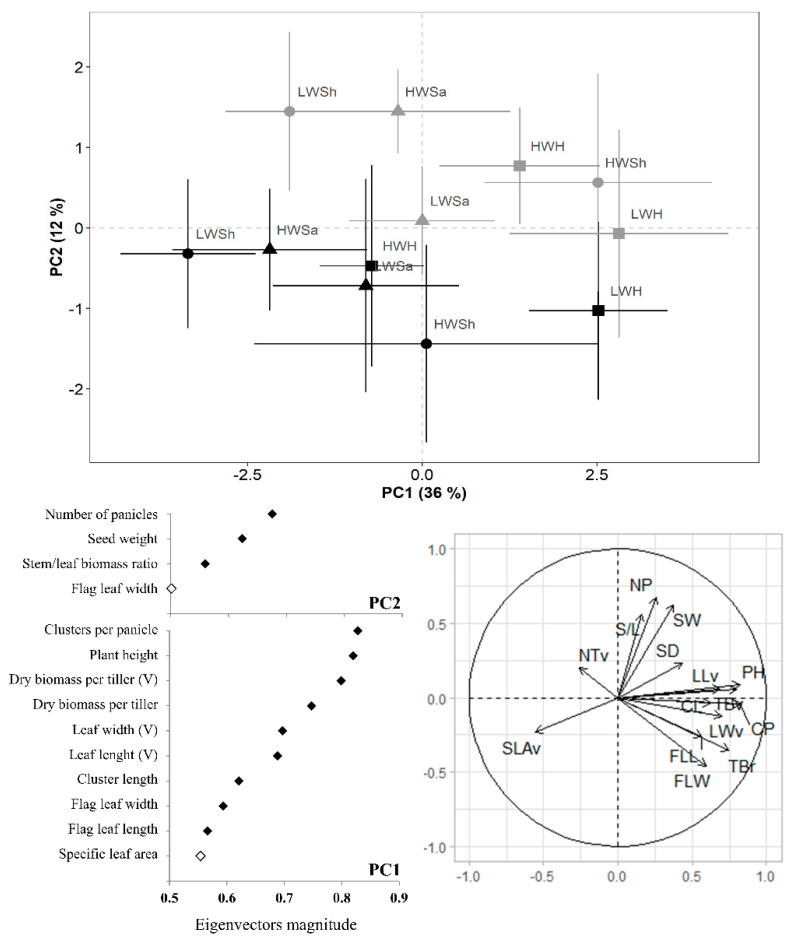
Principal Component Analysis (PCA) of plant functional traits in populations of *Leptochloa pluriflora* cultivated in two experimental environments: Sumalao (grey symbols) and Esperanza (black symbols) during the 2014–2015 summer season. The ID of populations is composed of seed weight (high/low) and origin of population. HWH: high weight-humid origin. HWSh: high weight-subhumid origin. HWSa: high weight-semiarid origin. LWH: low weight-humid origin. LWSh: low weight-subhumid origin. LWSa: low weight-semiarid origin. PH: Plant height. NP: Number of panicles. S/L: Stem/leaf biomass ratio. TBr: tiller biomass at the reproductive stage. SW: Seed weight. FLL: Flag leaf length. FLW: Flag leaf width. CP: Number of clusters per panicle. CL: Cluster length. NTv: Number of vegetative tillers. TBv: tiller biomass at the vegetative stage. LLv: Leaf length. LWv: Leaf width. SLAv: Specific leaf area.

In the PCA involving *L. pluriflora*, the two first components accounted for 48% of the total variance of the dataset (Figure 4). As found for *L. crinita*, PC2 (12%) sorted the populations according to the experimental environments, although less evidently than in *L. crinita*. This grouping was mainly due to reproductive features, such as number of reproductive tillers, seed weight, stem/leaf biomass ratio and flag leaf width. In Esperanza, plants produced lighter seeds, fewer reproductive tillers, lower stem/leaf biomass ratio and wider flag leaves. On the other hand, PC1 (36%) sorted the populations within each environment according to the features shown in Figure 4, also less clearly than in *L. crinita*. Populations from the same origin were grouped, but not in all cases. The specific leaf area (located in the lower left quadrant) was negatively correlated with almost all traits located in the right quadrant (except for number of vegetative tillers and stem/leaf biomass ratio). Seed weight was positively correlated with many reproductive traits related to the clusters, stem and flag leaf (Figure 4). See correlation matrix in Table S4.

## 3. Discussion

Our findings show a strong genetic control of seed weight in both false Rhodes grass species; this control is more evident in *L. crinita*, because no correlation was observed in seeds of *L. pluriflora* harvested in Esperanza in 2015. This phenomenon was already proven by Marinoni et al. [26] in an unstressed common garden. Here, no differences in seed weight related to maternal environment (arid and humid) were detected. Interestingly, the pattern between populations was almost the same in the arid and humid experimental environments, where populations maintained a stable seed weight which was even mirrored in the passport data, revealing a fixed ontogenetic variation [32]. This result suggests an adaptive genetic differentiation of the populations in both species caused by natural selection in the sites of origin [33,34,35,36]. In a previous work, we also associated seed weight variation in false Rhodes grass species with environmental variables of original habitats, which reinforces the idea of an adaptive trait [26]. 

The advantage conferred by heavy seeds in germination and seedling growth and survival has been widely documented [37,38,39,40,41]. The absence of germination of seeds of populations from arid and saline habitats under osmotic stress and the lack of interactions found in this work show that seed weight plays a more important role in germination behaviour than the original habitat of populations or the maternal environment. This fact is particularly important in forage grass breeding because of the low endosperm/embryo ratio in the seeds [42]; heavier seeds result in bigger seedlings, which in turn benefits plant growth, an important aim in forage breeding programmes [43]. Here, we found that heavier-seeded populations of both species produced heavier seedlings instead of a higher number of germinated seeds or higher germination rates. However, a weaker pattern for seed weight and germination behavior was found in *L. pluriflora* than in *L. crinita*; this weaker pattern might be due to two possible reasons: the high ploidy level of the former species [44], since higher ploidy levels increase the flexibility in the organism’s responsiveness to environmental changes [45,46,47], or the multiflower spikelet, in which resource allocation among flowers can vary with resource availability and fertility [48]. 

As already mentioned, heavier seeds provide advantages at early plant stages; however, at subsequent stages, other traits might be responsible for growth and development, either under stressful or optimal conditions. In the present study, fixed and plastic responses of functional traits were found in both species. While some traits varied in both experimental sites, other traits had a fixed behaviour, playing a major role in the differentiation patterns between populations within environments. In *L. crinita*, most of vegetative traits were plastic, whereas almost all reproductive traits showed a fixed response as the main driver of differentiation between populations. Plants grown under arid conditions (Sumalao) had more vegetative tillers and biomass per tiller, and longer leaves at the vegetative stage, but a reduced specific leaf area, stem diameter and dry biomass per tiller at the reproductive stage. This response reveals not only phenotypic plasticity for these parameters but also a trade-off strategy, whereby plants under arid conditions tend to produce more tillers and biomass per tiller, and longer leaves, reducing the specific leaf area during the vegetative stage; then, at the reproductive stage, leaf production is reduced to prioritise seed production and this reduction is also likely a sign of senescence.

In *L. crinita* plants subjected to experimental drought conditions, Quiroga et al. [15] found a decrease in leaf elongation rates and an increase in senescent leaves as the experiment progressed, which caused a decrease in the total biomass production. However, they found an adaptive pattern in specific leaf area related to differential responses of genotypes to drought conditions, whereas in the present work, such a trait was found to play a key role in the performance of all populations in both environments, exhibiting a phenotypic plastic response. Moreover, Greco and Cavagnaro [49] found a decrease in leaf area and leaf biomass under water stress, independently of the origin of *L. crinita* materials. On the other hand, parameters such as plant height, seed weight, stem diameter and leaf-related traits play a key role in plant adaptations to environment [10,11], as can be seen in this study for populations of *L. crinita*. Populations from saline origin had higher stem/leaf biomass ratio and seed weight but smaller leaf area (due to shorter and narrowed leaves) and lower plant height than populations from humid origin. The performance of the HWA population was similar to that of the population from saline environments, whereas the performance of the LWA population was similar to that of humid origin populations. Similarly, higher dry mass of panicles was found by Greco and Cavagnaro [49] in *L. crinita* genotypes from the driest origin, suggesting greater resource allocation to reproduction under stressful conditions. The populations that show similar responses to environmental conditions, which have similar effects on the dominant ecosystem processes, are considered functional types [36]. The fixed functional traits identified are key to identifying the best plant resource in breeding programmes for stressful environments.

*Leptochloa pluriflora* plants produced heavier seeds, more panicles, higher stem/leaf biomass ratio and narrower flag leaves in Sumalao than in Esperanza; thus, these traits showed a plastic response in this species. This finding does not agree with much evidence that reveals a trade-off strategy between inflorescences or seed number and seed weight [50,51,52]. Here, plants of *L. pluriflora* that produce heavier seeds had more panicles, as revealed by correlations. Number of inflorescences per plant or seeds per inflorescence can increase fecundity, whereas seed weight tends to favour establishment and survival, with the three traits being involved in ecological fitness [53]. This response may be a strategy of multiflower false Rhodes grass to favour survival under arid conditions. Moreover, traits such as biomass per tiller, leaf area, specific leaf area, plant height and panicle anatomy show a constant pattern within environments, suggesting that they are fixed traits in the species. However, specific leaf area was negatively correlated with many traits, either fixed or plastic, such as number of panicles, seed weight, cluster per panicle and biomass per tiller. A previous study [26] evaluating ecotypes from the same populations of both species as the ones studied here showed that ecotypes of *L. pluriflora* were more susceptible to stress than *L. crinita* ecotypes, exhibiting more dead biomass and decrease in shoot production, which was less evident in ecotypes from arid origins. However, these parameters were evaluated at the initial growth stage and under experimental conditions. There are no previous studies of *L. pluriflora* under water stress; only agronomic traits in common garden experiments or under field conditions have been studied [54,55,56,57]. 

Plastic and fixed responses were more evident in *L. crinita*. Populations showed a similar pattern in both experimental environments, except for populations from arid origins (LWA, HWA). In *L. pluriflora*, fewer trade-offs were found; this finding may be due to the higher ploidy level of this species, which can contribute to phenotypic plastic responses—as mentioned above—since only populations from the semiarid origin (LWSa, HWSa) had similar behaviour in both environments. As mentioned by Westoby [10], a leaf–height–seed scheme can be useful to evaluate plant ecological strategies. However, although plant height, specific leaf area and seed weight were correlated with many traits in the present study, we think that the inclusion of only a few traits may not be suitable to assess adaptive patterns at intraspecific level, where differences might be small. Although patterns were detected, the multivariate analysis did not explain much of the observed variation. Future studies using clones and/or epigenetic approaches may yield conclusive results. 

The results also suggest that considering environmental context instead of phylogeny [11,13] provides the most robust results in the selection of the most important plant functional traits. In addition to the ecological relevance, the information generated in the present study provides the basis for selecting seed sources in restoration plans or for pre-breeding for forage purposes in false Rhodes grass species. From the breeding point of view, the selection process can be slow when plastic responses are predominant. We identified populations with high biomass production and the traits that can be negatively affected in the selection process.

## 4. Materials and Methods

### 4.1. Trial 1. Seed Weight and Germination Analysis

#### 4.1.1. Plant Material

In a previous study [25], seed weight was evaluated in a common garden with accessions of *Leptochloa crinita* and *L. pluriflora* belonging to the germplasm bank “Ing. Agr. José M. Alonso” of the Facultad de Ciencias Agrarias of the Universidad Nacional del Litoral (FCA-UNL). For the present study, six accessions of the studied species were selected; they corresponded to populations of each species from different habitat types and with different seed weight (i.e., within each habitat type, a population with high seed weight and another with low seed weight were selected; Table 4). Seeds from each population (six per species) were obtained in 2013 from the aforementioned germplasm bank collection. Each population was represented by 10 randomly selected plants.

#### 4.1.2. Experimental Environments

Seeds of the selected populations were seeded in 1-L pots filled with a mixture of fertile soil and sand (3:1) during the first week of October 2013 (spring season). They were placed in a greenhouse under optimum conditions of light, humidity and temperature until plants reached three to four leaves (December 2013). At this stage, plants were transplanted to two climatically contrasting sites: the experimental field of the Estación Experimental Agropecuaria of the Instituto Nacional de Tecnología Agropecuaria in Sumalao, Valle Viejo, Catamarca (hereafter, S) and the experimental field of the FCA-UNL in Esperanza (hereafter, E), Santa Fe. Localities are 600 km apart and have similar average temperatures, but differ largely in annual rainfall (see Table S5). The experimental sites “S” and “E” were considered an arid and a humid site, respectively. Experimental environments were defined by localities and years (2 localities × 2 years = 4) because rainfall can vary between years. At each site, 120 plants of both species (2 species × 6 populations per species × 10 plant per population = 120) were arranged at a distance of 50 cm from each other in a Completely Randomized Design (CRD). Plants were grown and evaluated during two growing cycles (2014 and 2015). 

#### 4.1.3. Seed Weight Analysis

All mature panicles per plant were harvested from February to March in 2014 and 2015, and then threshed. Three samples of 1000 seeds (naked caryopsis)—from all harvested seeds per plant—were weighed using a precision balance (0.0001 g) to calculate the average seed weight per plant. Each plant represented a sample of the population.

#### 4.1.4. Germination Analysis

A germination analysis was performed in June 2015 using the seeds harvested from the four experimental environments: FCA-UNL Esperanza (site E) and EEA-INTA Sumalao (site S) during 2014 and 2015 (E-2014, S-2014, E-2015 and S-2015), considered maternal environments. Seeds collected in 2014 were stored under optimal conditions of temperature and humidity (4 °C at 14% RH) in a germplasm bank chamber until the start of the assays. There is evidence of absence of dormancy in seeds immediately after harvest [26,41]. Bulk samples of seeds were used for each population (i.e., seed pool generated by an equal proportion of seeds of each one of 10 plants representing the population). Seeds were subjected to control and osmotic stress conditions during germination. Osmotic stress was simulated by adding sodium chloride (NaCl) to sterile distilled water at the concentration of 120 mM per L of solution. This concentration of NaCl generates an osmotic effect in the short term [58]; by contrast, a polyethylene glycol (PEG) solution at the same osmotic pressure can make oxygen transport difficult [59]. Control consisted of sterile distilled water. For each species, three replicates of 30 seeds were used per population (6), experimental environment (4) and stress treatment (2). Replicates were randomly arranged in containers (8 × 3 × 3 cm) with a mesh at the bottom and filled with perlite. In each container, 30 mL of solution was added at the beginning of the trials; to avoid evapotranspiration, the solution was maintained in a humid environment in a germination chamber at 28 °C in the dark [26]. Before sowing in the containers, seeds were disinfected with sodium hypochlorite (10%) for two minutes and then rinsed three times with sterile distilled water. Moreover, viability of seeds was evaluated in a sample of 50 seeds of each population harvested from each experimental environment using the tetrazolium test [60]. Maguire’s germination rate (GR) [61], final emergence percentage (FEP) and seedling fresh mass (SFM) were calculated. The Maguire’s formula gives more importance to seeds germinated during earlier days than to those germinated during later days. The FEP was evaluated after 21 days of sowing [41] and was calculated based on viable seeds. The SFM was averaged from five seedlings weighed 21 days after sowing. In a previous study, highly significant correlations were found between initial and final emergence percentage and between fresh and dry mass of seedlings [26]. Therefore, in the present study, only one component of each pair was evaluated: FEP and SFM.

#### 4.1.5. Data Analysis

An Analysis of Variance (ANOVA, *p* < 0.05) was performed to evaluate seed weight variation among populations (6) and environments (4), and the interaction; normality and homoscedasticity assumptions were checked prior to statistical analysis. Data were analysed for each species separately. Mean differences were analysed by LSD test (*p* < 0.05). Additionally, correlations (Pearson, *p* < 0.05) between weight of seeds from different experimental sites and seed weight of passport data were assessed. Differences in germination variables—GR, FEP and SFM—were also evaluated by ANOVA (*p* < 0.05) for each species. The sources of variation were: population (6), experimental environment (4) and stress level (2). Mean differences were analysed by LSD test (*p* < 0.05). Correlations (Pearson, *p* < 0.05) between seed weight and germination variables were also assessed. R software [62] was used for the statistical analysis and the ggplot2 package [63] for plotting.

### 4.2. Trial 2. Plant Functional Traits

#### 4.2.1. Plant Material and Biometric Analyses

Since plants are exposed to stresses during and after transplanting, evaluation of functional traits in the first growing season may not be representative. Since both species are perennial, plants were cut after the first growing season and biometric measurements were made at both sites only during the second growing season. Plant functional traits were evaluated at vegetative and reproductive stages. Vegetative measurements were taken in individual plants in early summer, whereas reproductive features were evaluated in mid-summer. The following traits were measured per plant at the vegetative stage: number of tillers per plant, average dry biomass per tiller (g), average length and width of leaf ((cm) of the last fully expanded leaf) and average specific leaf area (cm^2^/mg). At the reproductive stage, the features evaluated were: plant height ((cm) measured in the tallest panicle), number of panicles per plant (same as reproductive tillers per plant), average dry biomass per tiller (g), average stem/leaf dry biomass ratio, average length and width of flag leaf (cm), average number of clusters per panicle, average length of cluster (cm) and seed weight (g/1000 seeds). Average values were estimated from three randomly selected tillers per plant. To estimate dry biomass, plant portions (i.e., leaves, stems, tillers) were oven-dried at 60 °C until constant weight.

#### 4.2.2. Data Analysis

Vegetative and reproductive traits for each species were subjected to a Principal Component Analysis (PCA). This multivariate analysis allows us to evaluate the implication of multiple functional traits in population performance in the two experimental environments. We also assessed bivariate correlations (Pearson, *p* < 0.05) with the purpose of identifying trade-offs among traits. Statistical analyses and plots were performed with R software [62] using the packages FactoMineR [64] and tidyverse.

## 5. Conclusions

In the present study we elucidated the adaptive role of seed weight in false Rhodes grass species. Each population of both false Rhodes grasses has a seed weight that is ontogenetically fixed and that can vary slightly under different aridity conditions; therefore, this trait is certainly stable. Our findings indicate that seed weight is a fixed adaptive trait involved in the germination process, favouring establishment and survival even under stressful conditions, while other functional traits gain importance at advanced growth stages. In *Leptochloa crinita*, the negative relationships of seed weight and specific leaf area with many vegetative traits provide evidence of trade-offs and resource allocation strategies, whereas in *L. pluriflora* some relationships were unexpected, such as the positive one between seed weight and number of panicles. Moreover, specific leaf area was negatively correlated with many traits, including seed weight, contrarily to findings in *L. crinita*. Our findings allowed us to detect the most important functional traits for the design of plant breeding programmes in false Rhodes grass species for drought stress tolerance.

## Figures and Tables

**Table 1:** Pearson’s correlation coefficients (r) for seed weight (SW) of populations of *Leptochloa crinita* and *L. pluriflora* harvested from two cultivation sites in two consecutive years. Probability values (*p*-values) are shown in parentheses.

***Leptochloa crinita***				
**Cultivation site ^1^**	**Original SW**	**E-2014**	**C-2014**	**E-2015**
**S-2015**	0.80 (<0.0001)	0.76 (<0.0001)	0.81 (<0.0001)	0.88 (<0.0001)
**E-2015**	0.81 (<0.0001)	0.81 (<0.0001)	0.81 (<0.0001)	
**S-2014**	0.76 (<0.0001)	0.82 (<0.0001)		
**E-2014**	0.78 (<0.0001)			
***Leptochloa pluriflora***				
**Cultivation site ^1^**	**Original SW**	**E-2014**	**C-2014**	**E-2015**
**S-2015**	0.73 (<0.0001)	0.32 (0.0150)	0.65 (<0.0001)	0.14 (0.1400)
**E-2015**	0.09 (0.5200)	0.27 (0.0430)	0.11 (0.4300)	
**S-2014**	0.56 (<0.0001)	0.43 (0.0010)		
**E-2014**	0.48 (0.0002)			

^1^ Esperanza site, year 2014 (E-2014), year 2015 (E-2015); Sumalao site, year 2014 (S-2014), year 2015 (S-2015).

**Table 2:** Mean values (±standard error) of final emergence percentage (FEP), seedling fresh mass (SFM) and Maguire’ germination rate (GR) for *Leptochloa crinita* populations and maternal environment (ME) of seeds evaluated under osmotic and non-osmotic stress.

		Control			Osmotic Stress		
ID Pop ^1^	ME	FEP	SFM	GR	FEP	SFM	GR
**LWA**	**S-2014**	27 ± 7 *a*	1.3 ± 0.2 *a*	5 ± 1 *a*	0 ± 0 *a*	0 ± 0 *a*	0 ± 0 *a*
	**S-2015**	34 ± 6 *a*	1.2 ± 0.3 *a*	5 ± 2 *a*	0 ± 0 *a*	0 ± 0 *a*	0 ± 0 *a*
	**E-2014**	39 ± 9 *a*	1.1 ± 0.3 *a*	6 ± 2 *a*	0 ± 0 *a*	0 ± 0 *a*	0 ± 0 *a*
	**E-2015**	38 ± 7 *a*	1.2 ± 0.3 *a*	7 ± 1 *a*	7 ± 1 *b*	1.1 ± 0.1 *b*	0.2 ± 0.1 *b*
**HWA**	**S-2014**	62 ± 9 *a*	2 ± 0.2 *ab*	12 ± 2 *a*	19 ± 0 *a*	1.7 ± 0.4 *ab*	2 ± 0 *a*
	**S-2015**	60 ± 11 *a*	1.6 ± 0.3 *a*	10 ± 1 *a*	21 ± 7 *a*	1.3 ± 0 *a*	1 ± 0 *a*
	**E-2014**	98 ± 1 *b*	2.4 ± 0.2 *b*	18 ± 0.5 *b*	34 ± 11 *b*	1.8 ± 0.1 *ab*	4 ± 0 *a*
	**E-2015**	53 ± 4 *a*	2.1 ± 0.3 *ab*	9 ± 0.5 *a*	27 ± 2 *ab*	2.1 ± 0.4 *b*	4 ± 0 *a*
**LWS**	**S-2014**	43 ± 1 *ab*	1.1 ± 0.2 *a*	8 ± 0 *bc*	0 ± 0 *a*	0 ± 0 *a*	0 ± 0 *a*
	**S-2015**	29 ± 0 *a*	0.9 ± 0.3 *a*	4 ± 1 *a*	0 ± 0 *a*	0 ± 0 *a*	0 ± 0 *a*
	**E-2014**	45 ± 8 *ab*	1.1 ± 0.1 *a*	6 ± 2 *ab*	0 ± 0 *a*	0 ± 0 *a*	0 ± 0 *a*
	**E-2015**	59 ± 11 *b*	1.3 ± 0.2 *a*	11 ± 2 *c*	0 ± 0 *a*	0 ± 0 *a*	0 ± 0 *a*
**HWS**	**S-2014**	50 ± 7 *a*	1.3 ± 0.2 *a*	8 ± 3 *a*	9 ± 1 *a*	1.5 ± 0.1 *b*	0.4 ± 0 *a*
	**S-2015**	68 ± 4 *a*	1.2 ± 0.3 *a*	13 ± 1 *a*	10 ± 1 *a*	0.9 ± 0.2 *a*	1 ± 1 *a*
	**E-2014**	45 ± 11 *a*	1.4 ± 0.1 *a*	8 ± 2 *a*	18 ± 2 *b*	0.8 ± 0.1 *a*	2 ± 1 *ab*
	**E-2015**	60 ± 4 *a*	1.7 ± 0.2 *a*	11 ± 0.5 *a*	37 ± 2 *c*	1.8 ± 0.1 *b*	4 ± 1 *b*
**LWH**	**S-2014**	46 ± 4 *a*	1.2 ± 0.4 *ab*	8 ± 1 *a*	11 ± 1 *a*	1.2 ± 0.1 *ab*	1 ± 0 *ab*
	**S-2015**	48 ± 5 *a*	0.8 ± 0.3 *a*	8 ± 1 *a*	8 ± 0 *a*	0.8 ± 0 *a*	1 ± 0 *ab*
	**E-2014**	34 ± 5 *a*	0.9 ± 0.1 *ab*	6 ± 2 *a*	26 ± 3 *b*	1.6 ± 0.3 *b*	3 ± 2 *b*
	**E-2015**	36 ± 5 *a*	1.8 ± 0.1 *b*	6 ± 3 *a*	10 ± 0 *a*	0.6 ± 0 *a*	0.3 ± 0.1 *a*
**HWH**	**S-2014**	12 ± 2 *a*	1.1 ± 0.1 *ab*	2 ± 0.5 *a*	7 ± 0 *a*	1 ± 0.1 *a*	1 ± 0.3 *b*
	**S-2015**	37 ± 4 *b*	1.5 ± 0 *b*	6 ± 1 *b*	6 ± 0 *a*	1.1 ± 0.1 *a*	0.3 ± 0 *a*
	**E-2014**	49 ± 7 *b*	1 ± 0.1 *a*	8 ± 1 *b*	12 ± 0 *ab*	1.6 ± 0 *b*	1 ± 0 *b*
	**E-2015**	49 ± 10 *b*	1.4 ± 0.2 *ab*	7 ± 1 *b*	17 ± 3 *b*	1.6 ± 0.4 *b*	1 ± 0.1 *b*

^1^ “ID pop” is composed of seed weight (high/low) and origin of population. HWA: high weight-arid origin. HWH: high weight-humid origin. HWS: high weight-saline origin LWA: low weight-arid origin. LWH: low weight-humid origin. LWS: low weight-saline origin. Different letters indicate statistically significant differences in seed weight between populations within each origin (LSD Fisher, *p* < 0.05).

**Table 3:** Mean values (±standard error) of final emergence percentage (FEP), seedling fresh mass (SFM) and Maguire’ germination rate (GR) for *Leptochloa pluriflora* populations and maternal environment (ME) of seeds evaluated under osmotic and non-osmotic stress.

		Control			Osmotic Stress		
ID Pop ^1^	ME	FEP	SFM	GR	FEP	SFM	GR
**LWSa**	**S-2014**	33 ± 6 *a*	1.8 ± 0.2 *b*	5 ± 0.5 *a*	6 ± 0 *b*	0.6 ± 0 *b*	1 ± 0.4 *b*
	**S-2015**	68 ± 7 *b*	1.3 ± 0.1 *a*	11 ± 1 *b*	10 ± 1 *c*	0.6 ± 0 *b*	0.5 ± 0.3 *ab*
	**E-2014**	86 ± 2 *c*	1.5 ± 0.1 *ab*	13 ± 2 *b*	0 ± 0 *a*	0 ± 0 *a*	0 ± 0 *a*
	**E-2015**	15 ± 0 *a*	1.5 ± 0.1 *ab*	2 ± 1 *a*	0 ± 0 *a*	0 ± 0 *a*	0 ± 0 *a*
**HWSa**	**S-2014**	95 ± 0 *b*	1.4 ± 0.1 *a*	18 ± 1 *b*	28 ± 3 *b*	1.2 ± 0.2 *ab*	3 ± 2 *ab*
	**S-2015**	97 ± 3 *b*	1.2 ± 0.2 *a*	18 ± 1 *b*	34 ± 4 *b*	1.4 ± 0 *b*	2 ± 1 *ab*
	**E-2014**	65 ± 4 *a*	1.3 ± 0.3 *a*	11 ± 1 *a*	34 ± 5 *b*	1.3 ± 0.2 *ab*	4 ± 2 *b*
	**E-2015**	51 ± 8 *a*	1.1 ± 0.2 *a*	8 ± 3 *a*	13 ± 3 *a*	0.8 ± 0.2 *a*	1 ± 0.1 *a*
**LWSh**	**S-2014**	52 ± 9 *a*	1.5 ± 0.2 *ab*	10 ± 2 *ab*	13 ± 4 *ab*	1.4 ± 0.2 *b*	1 ± 1 *a*
	**S-2015**	90 ± 4 *b*	1.4 ± 0 *ab*	17 ± 1 *c*	19 ± 9 *ab*	1.4 ± 0 *b*	1 ± 0.4 *a*
	**E-2014**	83 ± 8 *b*	2.1 ± 0.4 *b*	15 ± 2 *bc*	29 ± 8 *b*	1.6 ± 0.2 *b*	2 ± 1 *a*
	**E-2015**	58 ± 12 *a*	1.3 ± 0.3 *a*	8 ± 3 *a*	0 ± 0 *a*	0 ± 0 *a*	0 ± 0 *a*
**HWSh**	**S-2014**	62 ± 1 *b*	2.1 ± 0.4 *b*	11 ± 1 *b*	26 ± 10 *b*	1.5 ± 0 *b*	1 ± 0.5 *a*
	**S-2015**	100 ± 0 *c*	1.8 ± 0.5 *b*	19 ± 1 *c*	45 ± 11 *b*	1.4 ± 0.2 *b*	2 ± 1 *a*
	**E-2014**	74 ± 9 *b*	1.9 ± 0.2 *b*	13 ± 3 *b*	35 ± 8 *b*	1.8 ± 0.3 *b*	2 ± 1 *a*
	**E-2015**	26 ± 9 *a*	1.2 ± 0.1 *a*	4 ± 1 *a*	0 ± 0 *a*	0 ± 0 *a*	0 ± 0 *a*
**LWH**	**S-2014**	99 ± 1 *b*	1.6 ± 0.1 *b*	18 ± 1 *b*	31 ± 8 *ab*	1.1 ± 0.1 *ab*	3 ± 2 *ab*
	**S-2015**	100 ± 0 *b*	1.4 ± 0.2 *ab*	19 ± 1 *b*	52 ± 8 *b*	0.8 ± 0 *a*	3 ± 2 *ab*
	**E-2014**	86 ± 13 *b*	1.6 ± 0.4 *b*	16 ± 3 *b*	36 ± 6 *ab*	1.3 ± 0.5 *b*	4 ± 2 *b*
	**E-2015**	62 ± 9 *a*	1 ± 0.2 *a*	10 ± 3 *a*	10 ± 0 *a*	0.9 ± 0.2 *a*	0.5 ± 0 *a*
**HWH**	**S-2014**	100 ± 0 *b*	1.9 ± 0.2 *b*	19 ± 1 *b*	62 ± 6 *b*	1.9 ± 0.3 *b*	7 ± 4 *b*
	**S-2015**	100 ± 0 *b*	1.8 ± 0.2 *b*	19 ± 1 *b*	34 ± 8 *ab*	1.5 ± 0.1 *ab*	2 ± 0.3 *ab*
	**E-2014**	83 ± 8 *b*	1.4 ± 0.1 *a*	15 ± 2 *ab*	24 ± 0 *ab*	1 ± 0.1 *a*	2 ± 0.4 *ab*
	**E-2015**	68 ± 13 *a*	1.3 ± 0.2 *a*	12 ± 4 *a*	10 ± 1 *a*	1.2 ± 0.1 *a*	0.3 ± 0.2 *a*

^1^ “ID pop” is composed of seed weight (high/low) and origin of population. HWH: high weight-humid origin. HWSh: high weight-subhumid origin. HWSa: high weight-semiarid origin. LWH: low weight-humid origin. LWSh: low weight-subhumid origin. LWSa: low weight-semiarid origin. Different letters indicate statistically significant differences in seed weight between populations within each origin (LSD Fisher, *p* < 0.05).

**Table 4:** Origin and seed weight of *Leptochloa crinita* and *L. pluriflora* populations according to the accessions’ passport data of the *Leptochloa* collection of “Ing. Agr. José. M. Alonso” germplasm bank of the Facultad de Ciencias Agrarias of the Universidad Nacional del Litoral.

	*Leptochloa crinita*						*Leptochloa pluriflora*					
Accession ID	36	38	44	46	47	55	50	58	49	57	65	60
Province	Formosa	Santa Fe	San Luis	Córdoba	San Juan	Mendoza	Catamarca	San Luis	Salta	San Luis	Chaco	Santa Fe
Altitude (meters above sea level)	78	64	405	205	660	1173	426	590	736	466	104	84
Annual mean temperature (°C)	22.4	20.4	17.7	20.6	17.6	12.8	20.4	18	19.4	18.6	21.7	20.8
Annual rainfall (mm)	927	1140	452	515	178	318	388	504	638	607	968	847
Soil salinity (dS·m^−1^)	0.2	0.6	15.0	8.3	0.2	0.3	0.2	0.1	0.1	0.2	0.1	0.1
Origin classification (OC)	humid		saline		arid		semiarid		subhumid		humid	
Seed weight (SW, g/1000 seeds) ^1^	0.197 ^a^	0.242 ^b^	0.168 ^a^	0.205 ^b^	0.186 ^a^	0.353 ^b^	0.186 ^a^	0.250 ^b^	0.225 ^a^	0.250 ^b^	0.196 ^a^	0.244 ^b^
ID population (SW-OC)	LWH	HWH	LWS	HWS	LWA	HWA	LWSa	HWSa	LWSh	HWSh	LWH	HWH

^1^ Obtained in a common garden experiment [26]. Different letters indicate statistically significant differences in seed weight between populations within each origin (LSD Fisher, *p* < 0.05).

## Data Availability

Not applicable.

## Supplementary Materials

The following supporting information can be downloaded at: https://www.mdpi.com/article/10.3390/plants11212887/s1, Table S1: Two-way ANOVA (*p* < 0.05) table for seed weight variation analysis in *Leptochloa crinita* and *L. pluriflora*. Table S2: Probability values for ANOVA test from analysis of germination behaviour of populations of *Leptochloa crinita* and *L. pluriflora* (P) harvested from different maternal environments (ME) and evaluated under osmotic and non-osmotic germination treatments (GT). Table S3: Correlation coefficients and probability (*p*) values derived from Principal Component Analysis (PCA) performed for *Leptochloa crinita*. Table S4: Correlation coefficients and *p*-values derived from Principal Component Analysis (PCA) performed for *Leptochloa pluriflora*. Table S5: Characteristics of the experimental sites used for cultivation trials of the selected populations of *Leptochloa crinita* and *L. pluriflora*.
