# Supplementary material for: Seed Weight and Trade-Offs: An Experiment in False Rhodes Grasses under Different Aridity Conditions
Source: Plants (Basel). 2022 Oct 28;11(21):2887. doi: 10.3390/plants11212887 (PMC9654868; doi:10.3390/plants11212887)
Supplement: Supplementary file 1 [file plants-11-02887-s001.zip › plants-1917247-supplementary.pdf]

Table S1: The two-way ANOVA ($p<0.05$) table for seed weight variation analysis in *Leptochloa crinita* and *L. pluriflora.*

Leptochloa crinita

Variable	N	R^2	R^2 Aj	CV
Seed weight	194	0.83	0.8	10.21

Source of variation	Sum Sq	Df	Mean Sq	F-value	P-value
**Enviroments (E)**	0.016	3	5.00E-03	11.15	<0.0001
**Accessions (A)**	3.15E-01	5	6.30E-02	129.643	<0.0001
**E*A**	0.008	15	1.00E-03	1.124	0.3379
**Error**	0.084	172	4.90E-04		
**Total**	0.477	195			

Leptochloa pluriflora

Variable	N	R^2	R^2 Aj	CV
Seed weight	209	0.55	0.49	12.21

Soruce of variation	Sum Sq	Df	Mean Sq	F-value	P-value
**Enviroments (E)**	0.063	3	0.021	27.721	<0.0001
**Accessions (A)**	0.03	5	6.00E-03	7.836	<0.0001
**E*A**	0.041	15	3.00E-03	3.571	<0.0001
**Error**	0.147	193	1.00E-03		
**Total**	0.29	216			

Table S2: Probability values for ANOVA test from analysis of germination behaviour of populations of *Leptochloa crinita* and *L. pluriflora* (P) harvested from different maternal environments (ME) and evaluated under osmotic and non-osmotic germination treatments (GT).

	Leptochloa crinita						
	**ME**	**P**	**GT**	**ME * P**	**ME * GT**	**P * GT**	**ME * P * GT**
**Maguire' germination rate**	NS	<0.0001	<0.0001	0.0002	NS	0.0011	0.0001
**Final emergence percentage**	0.0006	<0.0001	<0.0001	0.0014	NS	0.0207	0.0039
**Seedling fresh mass**	<0.0001	<0.0001	<0.0001	NS	NS	<0.0001	0.0009
	Leptochloa pluriflora						
**Germination velocity**	<0.0001	<0.0001	<0.0001	0.0251	<0.0001	0.001	NS
**Final emergence percentage**	<0.0001	<0.0001	<0.0001	0.0478	NS	NS	NS
**Seedling fresh mass**	<0.0001	<0.0001	<0.0001	0.0001	NS	<0.0001	NS

Table S3: Correlation coefficients and probability (*P*) values derived from Principal Component Analysis (PCA) performed for *Leptochloa crinita*.

	PH	NP	S/L	TBr	SW	SD	FLL	FLW	CP	CL	NTv	TBv	LLv	LWv	SLAv
PH	1	0.0004	NS	0.0000	NS	0.0000	0.0000	0.0000	0.0000	0.0000	0.0025	0.0007	0.0001	0.0000	NS
NP	0.37	1	0.0100	NS	0.0200	NS	0.0100	NS	NS	NS	0.0400	0.0030	0.0000	0.0100	0.0300
S/L	-0.13	-0.28	1	0.0013	0.0000	NS	0.0000	0.0002	NS	0.0000	NS	0.0100	0.0000	0.0200	0.0007
TBr	0.48	-0.10	-0.33	1	NS	0.0000	0.0000	0.0000	0.0000	0.0000	NS	NS	NS	0.0000	NS
SW	-0.15	-0.24	0.72	-0.16	1	NS	0.0000	0.0200	NS	0.0001	0.0200	0.0042	0.0000	0.0035	0.0000
SD	0.48	-0.02	-0.19	0.79	-0.04	1	0.0000	0.0000	0.0000	0.0000	0.0600	NS	NS	0.0033	0.0100
FLL	0.58	0.29	-0.56	0.60	-0.55	0.52	1	0.0000	0.0000	0.0000	0.0200	0.0006	0.0000	0.0000	0.0100
FLW	0.60	0.11	-0.39	0.79	-0.24	0.73	0.70	1	0.0001	0.0000	NS	NS	0.0400	0.0000	NS
CP	0.44	-0.01	-0.08	0.59	-0.11	0.77	0.47	0.39	1	NS	NS	NS	NS	NS	NS
CL	0.41	0.21	-0.57	0.61	-0.39	0.51	0.64	0.70	0.12	1	NS	NS	0.0100	0.0000	NS
NTv	0.30	0.22	0.04	-0.08	-0.25	-0.20	0.25	0.02	0.05	-0.12	1	0.0000	0.0000	0.0000	0.0000
TBv	0.33	0.31	-0.27	0.07	-0.29	-0.08	0.34	0.18	-0.04	0.20	0.51	1	0.0000	0.0000	0.0000
LLv	0.37	0.41	-0.45	0.05	-0.66	-0.10	0.56	0.21	0.03	0.25	0.66	0.62	1	0.0000	0.0000
LWv	0.60	0.27	-0.24	0.44	-0.29	0.30	0.59	0.62	0.12	0.45	0.45	0.64	0.59	1	NS
SLAv	0.05	-0.23	0.35	0.16	0.59	0.27	-0.28	0.13	0.05	-0.07	-0.47	-0.42	-0.61	-0.08	1

PH: Plant height. NP: Number of panicles. S/L: Stem/leaf biomass ratio. TBr: tiller biomass at reproductive stage. SW: Seed weight. FLL: Flag leaf length. FLW: Flag leaf width. CP: Number of clusters by panicle. CL: Cluster length. NTv: Number of vegetative tillers. TBv: tiller biomass at vegetative stage. LLv: Leaf length at vegetative stage. LWv: Leaf width at vegetative stage. SLAv: Specific leaf area at vegetative stage.

Table S4: Correlation coefficients and *P*- values derived from Principal Component Analysis (PCA) performed for *Leptochloa pluriflora*.

	PH	NP	S/L	TBr	SW	SD	FLL	FLW	CP	CL	NTv	TBv	LLv	LWv	SLAv
PH	1	0.0027	NS	0.0000	0.0001	0.0000	0.0000	0.0000	0.0000	0.0000	NS	0.0000	0.0000	0.0000	0.0004
NP	0.28	1	NS	NS	0.0000	NS	NS	NS	NS	NS	0.0005	0.0100	0.0003	0.2200	0.0300
S/L	0.17	0.18	1	NS	0.0005	NS	NS	NS	NS	NS	NS	NS	NS	NS	NS
TBr	0.61	-0.04	0.00	1	NS	0.0300	0.0000	0.0000	0.0000	0.0000	NS	0.0000	0.0000	0.0000	0.0026
SW	0.37	0.39	0.33	0.06	1	0.0100	0.0044	NS	0.0100	0.0003	NS	0.0100	NS	NS	0.0000
SD	0.44	0.14	0.18	0.21	0.24	1	NS	NS	0.0000	0.0014	0.0400	0.0300	0.0200	NS	0.0048
FLL	0.43	0.01	-0.07	0.44	0.27	0.19	1	0.0000	0.0000	0.0000	NS	0.0100	0.0048	0.0400	0.0039
FLW	0.42	0.00	-0.10	0.55	-0.01	0.14	0.64	1	0.0000	0.0000	NS	0.0006	0.0001	0.0006	0.0300
CP	0.66	0.13	0.12	0.70	0.25	0.41	0.39	0.44	1	0.0002	0.0030	0.0000	0.0000	0.0000	0.0000
CL	0.47	0.11	0.11	0.49	0.33	0.30	0.55	0.44	0.34	1	NS	0.0000	0.0015	0.0100	0.0008
NTv	-0.18	0.33	-0.08	-0.17	-0.13	-0.20	-0.12	-0.11	-0.28	-0.14	1	NS	NS	NS	0.0100
TBv	0.56	0.24	0.10	0.59	0.26	0.21	0.25	0.32	0.69	0.37	-0.18	1	0.0000	0.0000	0.0000
LLv	0.57	0.34	0.11	0.45	0.08	0.21	0.27	0.35	0.52	0.29	0.05	0.69	1	0.0000	0.0100
LWv	0.56	0.12	0.06	0.58	0.08	0.13	0.20	0.31	0.56	0.25	-0.17	0.74	0.58	1	0.0100
SLAv	-0.32	-0.21	-0.08	-0.28	-0.37	-0.26	-0.27	-0.20	-0.49	-0.30	0.24	-0.51	-0.24	-0.24	1

PH: Plant height. NP: Number of panicles. S/L: Stem/leaf biomass ratio. TBr: tiller biomass at reproductive stage. SW: Seed weight. FLL: Flag leaf length. FLW: Flag leaf width. CP: Number of clusters by panicle. CL: Cluster length. NTv: Number of vegetative tillers. TBv: tiller biomass at vegetative stage. LLv: Leaf length at vegetative stage. LWv: Leaf width at vegetative stage. SLAv: Specific leaf area at vegetative stage.

Table S5: Characteristics of the experimental sites used for cultivation trials of the selected populations of *Leptochloa crinita* and *L. pluriflora*.

Feature	**FCA-UNL ESPERANZA (site *E*)**	**EEA-INTA SUMALAO (site S)**
Latitude (decimal degree)	-31.4424	-28.4724
Longitude (decimal degree)	-60.9410	-65.7297
Rainfall in the growing season* 2013/2014 (mm)	740**	337***
Rainfall in the growing season* 2014/2015 (mm)	767**	326***
Annual average temperature (°C)	18.9	20
Altitude (m above sea level)	38.8	518.4
Soil type	Typic Argiudoll	Typic Ustifluvent
Soil texture	Silty loam soil	Sandy soil

FCA-UNL: Facultad de Ciencias Agrarias of the Universidad Nacional del Litoral. **EEA-INTA:** Estación Experimental Agropecuaria of the Instituto Nacional de Tecnología Agropecuaria.

* From October to February. ** From the agrometeorological station of the FCA-UNL. *** From the agrometeorological station of the EEA-INTA San Fernando del Valle de Catamarca.
